# Retraction Note to: Analysis of risk factors related to the re-fracture of adjacent vertebral body after PKP

**DOI:** 10.1186/s40001-022-00739-3

**Published:** 2022-07-01

**Authors:** Shen-Yun Fang, Ji-Lin Dai, Ji-Kang Min, Wei-Li Zhang

**Affiliations:** 1 Orthopedics Department, The First People Hospital of Huzhou, The First People’s Hospital Affiliated to Huzhou Normal University, Huzhou, 313000 China; 2 Ophthalmology Department, The First People Hospital of Huzhou, The First People’s Hospital Affiliated to Huzhou Normal University, Huzhou, 313000 China

## Retraction to: Eur J Med Res (2021) 26:127 https://doi.org/10.1186/s40001-021-00592-w

The authors have retracted this article because it has been previously published by the same authors in a Chinese-language Journal [1]. Shen-Yun Fang, Ji-Lin Dai, Ji-Kang Min and Wei-Li Zhang have not responded to any correspondence from the editor/publisher about this retraction.
